# Micronests in Tumor Stroma is a Prognostic Classifier in Nasopharyngeal Carcinoma

**DOI:** 10.1097/PAS.0000000000002531

**Published:** 2026-03-16

**Authors:** Alhadi Almangush, Miia Ruuskanen, Jaana Hagström, Veli-Matti Kosma, Antti A. Mäkitie, Ilmo Leivo

**Affiliations:** * Department of Pathology; ‡ Research Program in Systems Oncology; # Research Programs Unit, Translational Cancer Medicine; §§ Research Program in Systems Oncology, University of Helsinki, Helsinki, Finland; ‡‡ Department of Otorhinolaryngology–Head and Neck Surgery, University of Helsinki and Helsinki University Hospital, HUS, Helsinki; † Institute of Biomedicine, Pathology; ∥ Department of Otorhinolaryngology–Head and Neck Surgery, Turku University Hospital; ¶ Department of Oral Pathology and Radiology; ¶¶ Institute of Biomedicine, Pathology, University of Turku and Turku University Central Hospital, Kiinamyllynkatu, Turku; ** School of Medicine, Institute of Clinical Medicine, Pathology and Forensic Medicine, and Cancer Center of Eastern Finland, University of Eastern Finland; †† Biobank of Eastern Finland, Kuopio University Hospital, Kuopio, Finland; § Libyan Authority for Scientific Research, Tripoli, Libya; ∥∥ Department of Clinical Sciences, Intervention and Technology, Division of Ear, Nose and Throat Diseases, Karolinska Institutet and Karolinska University Hospital, Stockholm, Sweden

**Keywords:** nasopharyngeal carcinoma, micronests in tumor stroma, survival

## Abstract

The assessment of micronests in tumor stroma has been introduced recently for the prognostication of human malignancies. In this multicenter study, we included a total of 110 patients treated for nasopharyngeal carcinoma (NPC) at one of the Finnish University Hospitals. All available hematoxylin and eosin (H&E)-stained slides were scanned. Assessment of micronests in tumor stroma was conducted on scanned HE-stained slides. Presence of micronests in tumor stroma had a significant prognostic value in predicting overall survival in the multivariable analysis with a hazard ratio (HR) of 1.92 (95% CI: 1.14-3.24, *P*=0.01). Similarly, in the multivariable analysis of disease-specific survival, micronests in tumor stroma had a significant prognostic value with an HR of 1.87 (95% CI: 1.00-3.48, *P*=0.04). In addition, the presence of micronests in tumor stroma showed a significant association with keratinizing and EBV-negative tumors (*P*<0.001). In conclusion, the evaluation of micronests in tumor stroma can be used as a prognostic classifier in NPC and they can be assessed in routine H&E-stained sections.

Nasopharyngeal carcinoma (NPC) has an aggressive behavior and the complex anatomic location further contributes to its challenging management. In addition, NPC has a unique geographic distribution with the majority of new cases arising in endemic regions in East and Southeast Asia.^1^ In nonendemic regions, however, NPC is a rare tumor and it has reportedly a higher rate of mortality compared with endemic NPCs.^2^ With regard to etiological factors, Epstein-Barr virus (EBV) infection has been closely associated with endemic NPC,^3,4^ while human papilloma virus (HPV) infection occurs in addition to EBV infection in nonendemic Western populations, in particular. Compared with the other head and neck cancers, NPC is associated with distinct clinical behavior including a high rate of lymph node metastasis, and there is a need for individualized treatments including de-escalation strategies for selected cases.^5,6^

The assessment of clinical behavior of NPC constitutes a critical challenge in daily practice. Despite the improvement in the therapeutic strategies, NPC is still a significant health issue in some countries.^7,8^ For decades, the main approach in the clinical decision-making of NPC is the classical TNM staging system, which lacks accuracy for many cases. Accurate risk stratification is a fundamental step during treatment planning. This includes specifically identification of patients who are at a high risk and would benefit from adjuvant therapy.^9^ Thus, there is a need for new prognostic indicators that are practical for further refinement of the staging system.^10^

Histopathologically, NPC is classified into keratinizing type, nonkeratinizing differentiated type, and nonkeratinizing undifferentiated type (which is the most common type in both endemic and nonendemic regions^11^). The assessment of histomorphological features of invading tumor cells/cell nests that characterize cancer cell dissociation has been associated with survival in many tumors.^12,13^ Micronests in tumor stroma have been defined as small clusters of cancer cells composed of 5 to 200 cancer cells or of a cluster of cancer cells <200 μm in diameter.^14^ Use of micronests in tumor stroma for prognostication of lung cancer has been introduced recently.^14^ The assessment method is simple and can be conducted in hematoxylin and eosin (H&E)-stained sections. In NPC, however, no information has been available about the clinical significance of micronests formation in tumor stroma. Therefore, this retrospective multicenter study aims to explore the prognostic significance of micronests formation in tumor stroma in a large multicenter cohort of NPC from a nonendemic region.

## MATERIAL AND METHODS

Inclusion criteria in this study were patients diagnosed with primary NPC with representative histologic specimens for prognostic evaluation. We excluded patients treated for other head and neck cancers, cases with insufficient histologic specimens, and patients treated with palliative intent. To ensure the histopathologic diagnosis of NPC, hematoxylin-eosin-stained specimens of all cases were re-evaluated by an experienced head and neck pathologist (I.L.). The diagnostic criteria used were those described in the fifth edition of the WHO classification of head and neck tumors.^15^ All cases were tested for EBV using in situ hybridization for EBV-encoded RNA (Ventana/Roche Medical Systems, Inc., Tucson, AZ), as described in our previous studies.^16,17^

A total of 169 cases treated for NPC were identified and their tumor blocks and clinicopathological characteristics were collected retrospectively. We excluded 54 cases because their samples were not representative (ie, insufficient tissue fragments), and 5 cases treated with palliative intent. The remaining 110 cases included in this study displayed representative samples with sufficient stromal areas in which we were able to assess the micronests in tumor stroma. These patients had been treated between 1990 and 2009 at one of the 5 Finnish University Hospitals (Helsinki, Turku, Tampere, Oulu, and Kuopio).

The number of tissue fragments available for each case ranged from one to 17 pieces with the majority of cases (93%) having at least 3 pieces. The width (ie, length) of the surface of the specimens varied from 1.4 to 13 mm with the majority of samples (80%) having a surface width of 3 mm or more. The depth of the samples varied from 0.8 to 11 mm with the majority of samples (87%) having a sample depth of 1.5 mm or more. Ethical permission for this research was obtained from the National Supervisory Authority for Welfare and Health (Valvira) and the Research Ethics Committee of the Hospital District of Southwest Finland.

**FIGURE 1:**
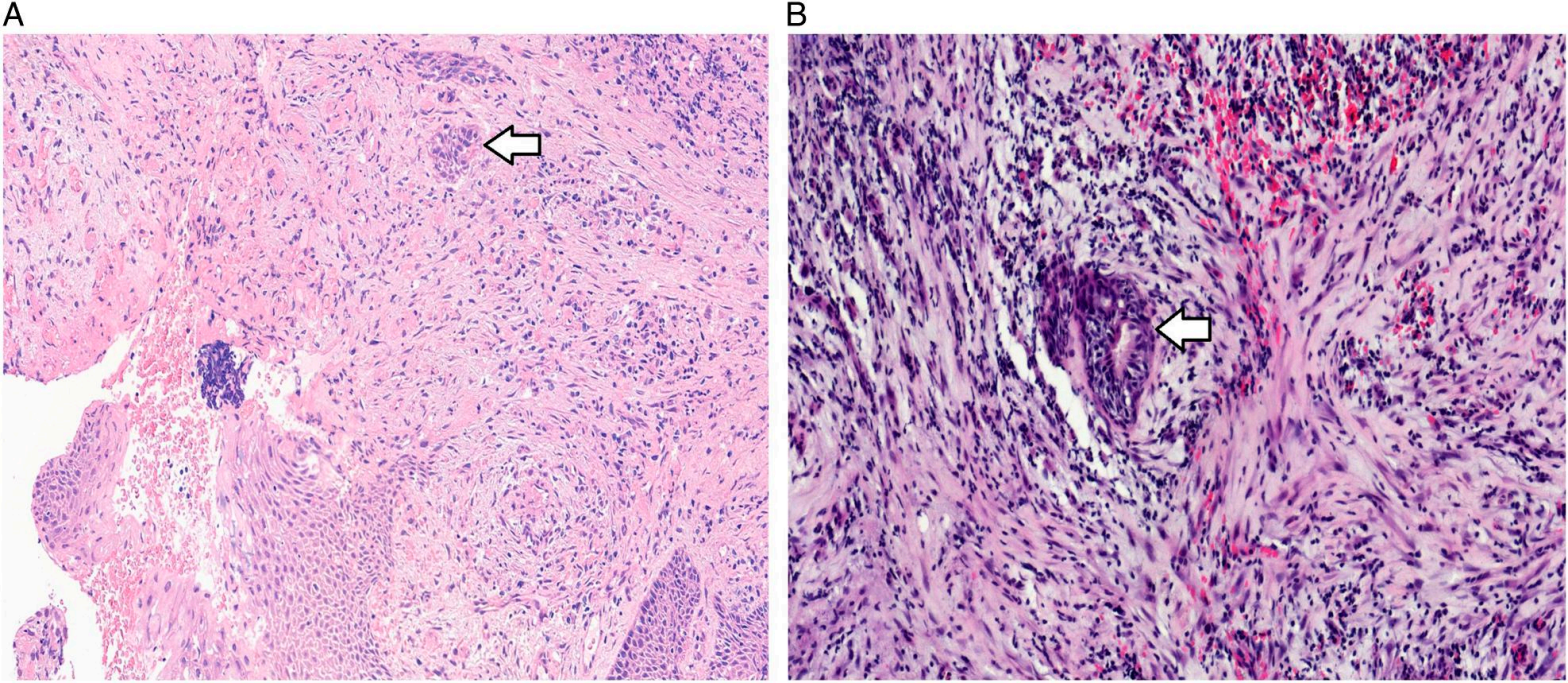
Histopathologic presentation of micronests in tumor stroma (arrow) in a hematoxylin and eosin-stained section of EBV-negative nasopharyngeal carcinoma (A). Micronests in tumor stroma (arrow) in EBV-associated nonkeratinizing nasopharyngeal carcinoma (B).

We assessed the presence of micronests in tumor stroma in HE-stained sections (Fig. 1). Micronests in tumor stroma have been defined as small clusters of cancer cells composed of 5 to 200 cancer cells or a cluster of cancer cells <200 μm in diameter.^14^ Micronests can be found anywhere in the tumor stroma, and they may be surrounded by a fibroblastic/collagenous stroma. In addition, they can be surrounded by inflammatory cell infiltrates. To ensure the applicability and to save the time, we scanned all slides, and images were generated using the 3DHISTECH Pannoramic 250 FLASH III digital slide scanner at Finnish Genome Editing Center supported by HiLIFE and the Faculty of Medicine, University of Helsinki, and Biocenter Finland. Measurement of the diameter of a micronests in tumor stroma was performed using a ruler tool of the 3DHistech CaseViewer software, that allows for a fast estimate of the tumor nest size, and after measuring, tumor nests larger than 200 μm in diameter were not considered.

Assessment of tumor-infiltrating lymphocytes (TILs) has been conducted in H&E-stained slides following the criteria of the International Immuno-Oncology Biomarkers Working Group^18^ as described in our previous study.^17^ In brief, the average amount of TILs was assessed within invasive tumors inside the tumor areas (ie, intratumoral TILs) and within the tumor stroma (ie, stromal TILs). A value for intratumoral TILs is given as the percentage of tumor islands/nests occupied by infiltrating lymphocytes, while the value for stromal TILs is given as the percentage of stromal areas occupied by infiltrating lymphocytes. The percentage of TILs were scored as incremental percentages (ie, 5%, 10%, 20%, 30%, 40%, etc). When tumors were classified as having “low TILs” or “high TILs”, 5% was deemed as an optimal cutoff point for intratumoral TILs, while for stromal TILs it was 10%, as described in our previous study.^17^

### Statistical Methods

We used SPSS 29 for all statistical analyses. We used cross-tabulation (Pearson χ^2^ test) to analyze the relationship between the micronests in tumor stroma and the clinicopathologic features of the respective patient (age, sex, stage, etc.). The relationship between micronests in tumor stroma and immune response (identified by the number of tumor-infiltrating lymphocytes that was previously evaluated in our research^17^), was also analyzed.

Survival endpoint prognostication included overall survival defined as the period from the end of the primary treatment to the date of death or the last follow-up visit; and disease-specific survival defined as the time from the end of the primary treatment to the time of death due to NPC. We reported hazard ratio (HR), 95% CI, and *P*-value (for both univariable and multivariable analyses) in each of the survival endpoints. In addition, Kaplan-Meier survival curves were reported for overall survival and disease-specific survival. Log-rank test was used to compare the survival curves.

## RESULTS

Of the 110 cases included, there were 75 (68.2%) men and 35 (31.8%) women with a ratio of men to women at 2.1, and a median follow-up time of 60 months. Histologic classification included 83 (75.5%) nonkeratinizing NPC tumors and 27 (24.5%) keratinizing NPC tumors. With regard to TNM staging, 15 (13.6%) tumors were stage I, 27 (24.5%) stage II, 38 (34.5%) stage III, and 30 (27.3%) stage IV. Fifty (45.5%) patients were treated with radiotherapy, while 60 (54.5%) patients were treated with chemoradiotherapy.

A total of 52 (47.3%) tumors showed micronests in tumor stroma. In cross-tabulation (Table 1), a significant correlation was observed between the presence of micronests in tumor stroma and keratinizing histology of tumors (*P*<0.001). Micronests in tumor stroma were significantly associated with EBV-negativity (*P*<0.001). Furthermore, micronests in tumor stroma showed a significant association with low tumor-infiltrating lymphocytes (*P*<0.05). No significant association was noted between micronests in tumor stroma and any other clinicopathologic factor (*P*>0.05).

**TABLE 1:** Correlation Between Micronests in Tumor Stroma and Clinicopathologic Features of 110 Cases Treated for Nasopharyngeal Carcinoma

		Micronests in tumor stroma		
	Total	None	Present	
Variable	N=110	Number (%) 58 (52.7%)	Number (%) 52 (47.3%)	*P* (Pearson χ^2^)
Age				0.94
≤60 y	66	35 (53.0)	31 (47.0)	
>60 y	44	23 (52.3)	21 (47.7)	
Sex				0.16
Male	75	43 (57.3)	32 (42.7)	
Female	35	15 (42.9)	20 (57.1)	
Stage				0.13
Early stage (I-II)	42	26 (61.9)	16 (38.1)	
Advanced stage (III-IV)	68	32 (47.1)	36 (52.9)	
EBV status				<0.001
Positive	68	45 (66.2)	23 (33.8)	
Negative	40	12 (30.0)	28 (70.0)	
Histology				<0.001
Nonkeratinizing	83	55 (66.3)	28 (33.7)	
Keratinizing	27	3 (11.1)	24 (88.9)	
Intratumoral TILs				<0.001
Low	35	4 (11.4)	31 (88.6)	
High	75	54 (72.0)	21 (28.0)	
Stromal TILs				<0.002
Low	29	8 (27.6)	21 (72.4)	
High	81	50 (61.7)	31 (38.3)	

**FIGURE 2:**
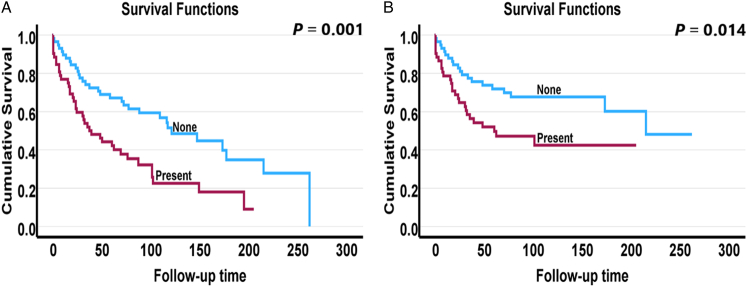
Kaplan-Meier survival curves for overall survival (A) and disease-specific survival (B) of 110 nasopharyngeal carcinoma cases with and without micronests in tumor stroma.

In survival analysis (Table 2), the presence of micronests in tumor stroma were associated with worse overall survival in univariable analysis with an HR of 2.16 (95% CI: 1.33-3.49, *P*=0.002), and in the multivariable analysis with an HR of 1.92 (95% CI: 1.14-3.24, *P*=0.01). In disease-specific survival, micronests in tumor stroma showed a powerful prognostic value with an HR of 2.05 (95% CI: 1.14-3.70, *P*=0.02) in the univariable analysis, and an HR of 1.87 (95% CI: 1.00-3.48, *P*=0.04) in the multivariable analysis. In addition, Kaplan-Meier survival curves (Fig. 2) were also constructed for the cases with micronests in tumor stroma present versus no micronests present, and they showed a significant relationship between the presence of micronests in tumor stroma and poor overall survival (*P*=0.001) as well as worse disease-specific survival (*P*=0.014).

**TABLE 2:** Overall Survival and Disease-Specific Survival Analyses in a Cohort of 110 Patients of Nasopharyngeal Carcinoma

	Overall survival				Disease-specific survival			
Parameter	Univariable analysis HR (95% CI)	*P*	Multivariable analysis HR (95% CI)	*P*	Univariable analysis HR (95% CI)	*P*	Multivariable analysis HR (95% CI)	*P*
Age		*P*=0.01		*P*=0.008		*P*=0.28		*P*=0.11
≤60	Reference		Reference		Reference		Reference	
>60	1.86 (1.16-2.99)		1.99 (1.20-.29)		1.38 (0.77-2.46)		1.62 (0.89-2.96)	
Sex		*P*=0.90		*P*=0.47		*P*=0.93		*P*=0.87
Male	Reference		Reference		Reference		Reference	
Female	0.97 (0.59-1.61)		0.82 (0.48-1.39)		1.03 (0.55-1.91)		1.05 (0.55-2.02)	
Stage		*P*=0.004		*P*=0.008		*P*<0.001		*P*<0.001
Early stage (I-II)	Reference		Reference		Reference		Reference	
Advanced stage (III-IV)	2.13 (1.27-3.57)		2.07 (1.21-3.54)		4.03 (1.88-8.66)		3.82 (1.75-8.36)	
EBV status		*P*<0.001		*P*<0.001		*P*=0.009		*P*=0.19
Positive	Reference		Reference		Reference		Reference	
Negative	3.37 (2.06-5.51)		2.47 (1.44-4.23)		2.21 (1.22-4.01)		1.53 (0.81-2.87)	
Micronests in tumor stroma		*P*=0.002		*P*=0.01		*P*=0.02		*P*=0.04
None	Reference		Reference		Reference		Reference	
Present	2.16 (1.33-3.49)		1.92 (1.14-3.24)		2.05 (1.14-3.70)		1.87 (1.00-3.48)	

Of note, in EBV-positive cases, those tumors that presented with micronests in tumor stroma were associated with worse overall survival (HR: 2.18, 95% CI: 1.09-4.35, *P*=0.027) and disease-specific survival (HR: 2.42, 95% CI: 1.08-5.41, *P*=0.032), compared with tumors without micronests in tumor stroma. In addition, in nonkeratinizing tumors the presence of micronests in tumor stroma identified a group of cases with a poor overall survival (HR: 2.03, 95% CI: 1.13-3.64, *P*=0.018) and worse disease-specific survival (HR: 2.06, 95% CI: 1.00-4.24, *P*=0.04).

## DISCUSSION

Predicting the prognosis of nasopharyngeal cancer is sometimes challenging due to heterogeneity of cases causing diverse clinical outcomes even in cases with the same stage.^19^ The current TNM staging system is insufficient for accurate prognostication and researchers are evaluating the possibility of incorporating other prognostic factors.^1^ Identification of biomarkers and developing of treatment strategies for NPC are mostly carried out in studies from endemic regions. In the current multicenter study of a nonendemic population, we found that the presence of micronests in tumor stroma is associated with poor prognosis of NPC. To the best of our knowledge, this is the first study to assess micronests in tumor stroma in NPC.

Dissemination of tumors occurs as single-cell and/or collective cell invasion.^20^ NPC is well-known as a highly infiltrative cancer.^10^ In head and neck cancers, noncohesive patterns of spread have been specifically associated with poor prognosis.^12,21,22^ Recently worst pattern of invasion in squamous cell carcinoma has been widely studied. However, the worst pattern of invasion has been criticized as some perineural and lymphovascular invasion might present as type 5.^22^ Furthermore, there is a lack of histopathologic parameter/s to assess the pattern of the invading tumor cells in NPC using H&E-stained sections. Therefore, micronests in tumor stroma that can be assessed in a simple procedure are a good choice for the characterization of invasive growth in NPC. The small size of biopsies of NPC may make it difficult to use other parameters, such as tumor budding defined as clusters of <5 cancer cells.^23^ Because of their larger size, micronests in tumor stroma appear to be more easily detectable than tumor budding.^14^ Moreover, tumor budding can be difficult to assess in the presence of a dense inflammatory cell infiltration,^14^ which is common in NPC.

In this study, we found that micronests in tumor stroma in NPC have a powerful association with patient survival. This is in line with previous research on other tumors in other locations.^14^ In addition, micronests in tumor stroma presented more often in keratinizing NPCs compared with nonkeratinizing. They also presented more often in EBV-negative tumors which are known to behave more aggressively than EBV-positive. One important finding in the current study was that the presence of micronests in tumor stroma identified a group of cases within the EBV-positive tumors with a worse survival. Furthermore, the presence of micronests in tumor stroma identified nonkeratinizing NPCs with poor survival. This indicates that in such cases, micronests in tumor stroma can be used to support clinical decision for the use of aggressive therapy. In contrast, the absence of micronests in tumor stroma suggests a favorable prognosis, and such cases might be considered for de-escalation treatment. However, precise prognostication for treatment planning (including de-escalation) should be based on multiple prognostic parameters.^24^ For example, in such cases, combining micronests in tumor stroma with other well-known prognostic biomarkers, such as the level of Epstein-Barr virus DNA^6^ improves risk stratification. Of note, combining prognostic markers has been recently introduced in NPC using a web-based prognostic tool built using machine learning algorithms that easily include multiple prognostic factors.^25^ However, this approach will require further research.

In conclusion, the relationship between micronests in tumor stroma and patient survival emphasizes the usefulness of this histopathologic feature in risk stratification of NPC. Furthermore, the association of micronests in tumor stroma with adverse prognostic indicators such as keratinizing histology and EBV-negativity, further highlights its prognostic value in NPC. The assessment is performed using routine H&E-stained sections and the results may potentially influence the selection of therapeutic choices. The findings of our study can aid in selecting those patients who require adjuvant chemotherapy as they are at high risk of poor survival. It is essential to further validate the present findings in other cohorts, preferably, in multi-institutional studies and of a prospective nature. In addition, for the assessment of micronests in tumor stroma, it is necessary to consider criteria for a representative biopsy. An insufficient superficial biopsy may lead to a failure in diagnosing NPC^26^ and precluding the assessment of prognostic indicators such as micronests in tumor stroma.
